# Visualization of atherosclerosis as detected by coronary artery calcium and carotid intima-media thickness reveals significant atherosclerosis in a cross-sectional study of psoriasis patients in a tertiary care center

**DOI:** 10.1186/s12967-016-0947-0

**Published:** 2016-07-22

**Authors:** S. Santilli, D. R. Kast, I. Grozdev, L. Cao, R. L. Feig, J. B. Golden, S. M. Debanne, R. C. Gilkeson, C. E. Orringer, T. S. McCormick, N. L. Ward, K. D. Cooper, N. J. Korman

**Affiliations:** Department of Dermatology, University Hospitals Case Medical Center, 11000 Euclid Ave, Cleveland, OH 44106 USA; The Murdough Family Center for Psoriasis, Cleveland, USA; Center For Clinical Investigation, Case Western Reserve University, Cleveland, USA; Department of Epidemiology and Biostatistics, Case Western Reserve University, Cleveland, USA; Department of Pathology, Case Western Reserve University, Cleveland, USA; Louis Stokes VA Medical Center, Cleveland, OH USA; University of Miami, Miller School of Medicine, Miami, FL 33125 USA

**Keywords:** Psoriasis, Cardiovascular disease, Vascular inflammation, Coronary artery calcium, Carotid intima-media thickness, Psoriatic arthritis, hs-CRP

## Abstract

**Background:**

Psoriasis is a chronic inflammatory disease of the skin and joints that may also have systemic inflammatory effects, including the development of cardiovascular disease (CVD). Multiple epidemiologic studies have demonstrated increased rates of CVD in psoriasis patients, although a causal link has not been established. A growing body of evidence suggests that sub-clinical systemic inflammation may develop in psoriasis patients, even from a young age. We aimed to evaluate the prevalence of atherosclerosis and identify specific clinical risk factors associated with early vascular inflammation.

**Methods:**

We conducted a cross-sectional study of a tertiary care cohort of psoriasis patients using coronary artery calcium (CAC) score and carotid intima-media thickness (CIMT) to detect atherosclerosis, along with high sensitivity C-reactive protein (hsCRP) to measure inflammation. Psoriasis patients and controls were recruited from our tertiary care dermatology clinic. Presence of atherosclerosis was defined using validated numeric values within CAC and CIMT imaging. Descriptive data comparing groups was analyzed using Welch’s t test and Pearson Chi square tests. Logistic regression was used to analyze clinical factors associated with atherosclerosis, and linear regression to evaluate the relationship between psoriasis and hsCRP.

**Results:**

296 patients were enrolled, with 283 (207 psoriatic and 76 controls) having all data for the hsCRP and atherosclerosis analysis. Atherosclerosis was found in 67.6 % of psoriasis subjects versus 52.6 % of controls; Psoriasis patients were found to have a 2.67-fold higher odds of having atherosclerosis compared to controls [95 % CI (1.2, 5.92); p = 0.016], after adjusting for age, gender, race, BMI, smoking, HDL and hsCRP. In addition, a non-significant trend was found between HsCRP and psoriasis severity, as measured by PASI, PGA, or BSA, again after adjusting for confounders.

**Conclusions:**

A tertiary care cohort of psoriasis patients have a high prevalence of early atherosclerosis, increased hsCRP, and psoriasis remains a risk factor for the presence of atherosclerosis even after adjustment of key confounding clinical factors. Psoriasis may contribute to an accelerated systemic inflammatory cascade resulting in increased risk of CVD and CV events.

**Electronic supplementary material:**

The online version of this article (doi:10.1186/s12967-016-0947-0) contains supplementary material, which is available to authorized users.

## Background

Psoriasis is a chronic inflammatory disease of the skin and joints that may also have systemic inflammatory effects, including the development of cardiovascular disease (CVD) [1]. While the cutaneous manifestations of psoriasis wax and wane the systemic inflammatory effects may incite continuous, progressive development of CVD and atherosclerosis [2–6]. Multiple epidemiological studies have demonstrated elevated rates of cardiovascular events in psoriasis patients when compared to controls [7–10]. From McDonald and Calabresi’s study of psoriasis and occlusive vascular disease in 1978 to Gelfand’s 2006 landmark study, many studies have also linked psoriasis with increased mortality specifically related to CVD [11, 12]. This finding has traditionally been explained by the higher prevalence of CVD risk factors in psoriasis patients, such as the components of the metabolic syndrome, tobacco, and alcohol abuse [13–15]. These confounding factors have led to debate as to whether or not psoriasis incurs independent risk for the development or progression of cardiovascular disease. There have been several studies that demonstrate no independent association between psoriasis and the development of atherosclerosis [15–17]. Proponents of the association between psoriasis and CVD support the concept that CVD risk factors favor inflammation and atherogenesis, and when combined with the pro-inflammatory state of psoriasis, a synergistic effect may result [4, 6, 18]. Indeed, murine models of psoriasiform skin have demonstrated that chronic skin inflammation can lead to vascular inflammation and increased rates of thrombosis, suggesting that chronic inflammation exacerbates cardiovascular complications [19]. Now, in a separate study, these observations have been extended to a second skin-contained transgenic mouse model, demonstrating that chronic, but not acute skin inflammation promotes arterial thrombosis [20].

Of utmost concern in psoriasis patients is the possibility of developing significant CVD at a relatively young age that potentially results from this synergistic pro-inflammatory milieu and the duration of exposure to this milieu. Large population-based studies demonstrate an increased incidence of CVD including stroke, especially among younger, severe psoriasis patients [21–24]. Several studies have even reported an increased risk of CV mortality with psoriasis [10, 25, 26]. Thus, these concerns have led to studies investigating the link between psoriasis and sub-clinical CVD using special imaging techniques. These techniques include coronary artery calcium scoring (CAC), carotid intimal media thickness (CIMT), and brachial artery flow-mediated dilation (FMD) amongst others. A systematic review by Shaharyar et al. [27] evaluated multiple studies using these techniques to assess sub-clinical atherosclerosis and concluded that in general, psoriasis patients had higher CIMT and CAC burden as well as endothelial dysfunction compared to controls. However, these studies have individual limitations that invite further investigation into a potential causal link between psoriasis and CVD. One other common link in the inflammatory cascade of psoriasis and CVD may be C-reactive protein (CRP). Extensively studied for its implication in CVD, CRP is regulated in the acute phase by IL-1, IL-6, and TNF-α [28, 29]. Synthesized primarily by the liver, CRP is also produced by coronary artery smooth muscle cells in response to inflammatory stimuli, and provides integration of overall cytokine activation [18, 28]. CRP has been shown to be associated with all-cause mortality in chronic immune-mediated inflammatory disease, including psoriasis [30] and to correlate with risk of cardiovascular events in patients who have instituted aggressive statin therapy [31]. Some studies suggest that CRP levels can predict prognosis in those with a cardiovascular event, and the high sensitivity test of CRP (hsCRP) may be predictive of cardiovascular events in asymptomatic, healthy populations [32–36], although other studies do not support this contention [37]. While its possible role in the genesis of CV events has not been elucidated, CRP may be an important indicator of cardiovascular risk.

Our aim was to evaluate the prevalence of atherosclerosis in a tertiary care psoriasis cohort and the association between psoriasis, atherosclerosis and inflammatory markers while controlling for major potential confounders. This objective was completed via a cross-sectional study using multi-modal vascular evaluation of the carotid arteries for the presence of carotid plaque, carotid intima-media thickness (CIMT), computed tomography (CT) of the coronary arteries for calcification (CAC), measurement of hsCRP, and measurement of CVD risk factors and psoriasis.

## Methods

### Subjects

After approval by the Institutional Review Board of University Hospitals Case Medical Center (UHCMC, Cleveland, OH, USA), psoriasis patients and control volunteers seen throughout the year in our tertiary care clinics (psoriasis diagnosed by NJK or KDC) ≥18 years were invited to participate.

### Psoriasis patients

#### Inclusion criteria

Psoriasis patients with and without a history of psoriatic arthritis were eligible. Psoriatic arthritis was assessed by asking patients if they had experienced joint pain or swelling, morning stiffness, had been diagnosed with psoriatic arthritis, or had been treated by a rheumatologist for psoriatic arthritis.

#### Exclusion criteria

Patients with a known or suspected history of systemic inflammatory diseases, with the exception of psoriatic arthritis, were excluded.

### Healthy control volunteers

#### Inclusion criteria

Controls were either: (1) Individuals recruited from the same dermatology clinics who were being seen for common dermatologic complaints including seborrheic keratoses, warts, nevi, and actinic keratosis; or (2) Non-genetically-related subjects residing with psoriasis patients.

#### Exclusion criteria

Control patients with a history of atopic dermatitis, contact dermatitis, acne, connective tissue disease or autoimmune-blistering diseases were excluded, as these conditions are known systemic inflammatory diseases (n = 62).

The control cohort is comparable to the psoriasis cohort as both populations are from the same geographical area and seek treatment for a dermatologic complaint, or reside with psoriasis patients. Approximately 20 % of screened psoriasis patients and healthy control volunteers declined to participate, citing time restraints, or unwillingness to be involved in a research protocol.

Eligible patients participated in a cross-sectional study visit having fasted for 8 h, refrained from exercise for 6 h, and refrained from vitamins, oral antioxidants, tobacco, caffeine and antihypertensive medication on the study date. We measured height, weight, blood-pressure (after being seated for 5 min, on both arms, then averaged), hip and waist circumference, psoriasis area severity index (PASI), Physician’s Global Assessment (PGA), and body surface area (BSA). Carotid plaque, CIMT and CAC were measured. Fasting venous blood was obtained for hsCRP, total cholesterol, high density lipoprotein (HDL), triglycerides, low density lipoprotein (LDL) as calculated by the Friedewald equation, and blood glucose which was quantified by the UHCMC clinical laboratory. In addition, medical history was completed including evaluation of known risk factors for CAD, including smoking history, history of hypertension, gender, and race. These were included as binary variables, analyzed as potential confounders, except for race, which was evaluated as a categorical variable. A medical history of atherosclerotic disease was obtained, including a history of coronary or peripheral vascular angioplasty or stenting, as well as history of angina or evidence of peripheral ischemia, and transient ischemic attack or stroke. These patients were included in the analysis of prevalence. All patients who agreed to participate were included in the study.

### Clinical measures

Ultrasound was performed using a Toshiba Nemio XG ultrasound machine (model #SSA-580A). We used a 9 MHz linear probe with a depth of 4 cm and a dynamic range set at 70 dB and 32 frames/s.

For CIMT, right and left distal common carotid arteries (CCA) were scanned at the anterior, lateral, and posterior angles. Each angle was imaged at 3 consecutive R-waves for a total of 9 images on each side. We used the Carotid Analyzer for Research software (Medical Imaging Applications, LLC, Coralville, IA, USA). Mean CIMT was measured from a 1 cm segment of the far wall of the CCA proximal to the bulb. The average values of the 3 images at each angle were averaged, yielding a mean of all 3 angles as the mean of means for each side. Carotid plaque, defined as encroachment into the vessel lumen ≥1.5 mm or thickening of greater than 50 % than the adjacent segments, was measured by the same technique scanning the length of both internal and external carotid arteries.

Ultrasound procedures were performed by one sonographer (RLF) to eliminate interpersonal technique differences and all measurements were completed by RLF and audited by an experienced vascular medicine specialist.

CAC scoring was assessed with non-contrast enhanced technique by one of two CT (Somatom Sensation 16; Siemens Inc., Malvern PA, USA) scanners performed using the following parameters: 140 kV, 30 mAs, B35f filter, 12 × 1.5 mm collimation, 3 mm reconstruction, 0.36 s scan time, 3.15 mGy average dose; Somatom Sensation 64, 120 kV, 40 mAs, B35f filter, 30 × 0.6 mm collimation, 3 mm reconstruction, 0.36 s scan time, 2 mGy average dose, in the Department of Radiology at UHCMC. Electrocardiogram leads were placed and heart rates were monitored. As long as the heart rate was <100 beats/min, the coronary arteries were able to be studied. Images were interpreted by one of three board-certified radiologists in the cardiothoracic imaging section of the Department of Radiology, UHCMC. CAC scoring was calculated according to the method of Agatston. The CT attenuation threshold for detection of coronary artery calcification was 130 Hounsfield units.

### Evidence of atherosclerosis

We defined atherosclerosis as a CAC score ≥1, or right or left CIMT >75th percentile, or carotid plaque. Patients with coronary stent(s) were defined as having a positive CAC score. Our definition uses validated techniques to diagnose atherosclerosis [38]. CIMT >75th percentile was determined by plotting the mean of the mean values of CIMT against data from the Atherosclerosis Risk in Communities study, which adjusts for age, gender, and race [39, 40].

### Statistical analysis

Demographics and disease characteristics of psoriasis and controls were summarized using means and standard deviations for continuous variables and frequencies and proportions for categorical variables. We used a two-sample Welch’s t test to compare mean levels and a Pearson’s Chi squared test to compare proportions between the two groups. The prevalence of atherosclerosis in the psoriasis cohort relative to controls was assessed using logistic regression models to estimate odds ratios.

To model the association between psoriasis and the prevalence of atherosclerosis, logistic regression models were used to estimate the odds ratio of atherosclerosis comparing psoriasis to controls. Multivariable logistic regression models adjusted for confounders including age, gender, race, BMI (weight lbs./(height in.)^2^ × 703), current smoking status, history of hypertension (systolic ≥140 mm Hg, or diastolic ≥90 mm Hg, or current use of anti-hypertensive medication), serum HDL, and hsCRP. Interactions were not evaluated. Sub-group analysis was performed to evaluate both patients with psoriasis and psoriatic arthritis. Adequacy of the models was assessed using the Hosmer and Lemeshow goodness-of-fit test.

Separate linear regression models were used to estimate the association between psoriasis and hsCRP. hsCRP was right skewed so a log-transformation was used to model the association between psoriasis and hsCRP. Multivariable linear regression models were used to estimate the geometric mean ratio of hsCRP comparing psoriasis to controls, adjusting for age, gender, race, BMI, current smoking status, history of hypertension, serum HDL, and the presence of atherosclerosis. Residuals were examined for model adequacy.

A similar modeling strategy was followed, using only psoriasis patients, to investigate the association between various measures of psoriasis severity and the prevalence of atherosclerosis, or hsCRP. A sensitivity analysis that excluded those patients with a medical history of cardiovascular disease was conducted. No adjustment for multiple comparisons was made. All analyses were based on a two-sided significance level of 0.05 and were performed using SAS 9.2 and 9.4 (SAS Institute, Cary, NC, USA). No a priori sample size calculations were performed.

## Results

### Study cohort

295 subjects were enrolled. 283 (207 psoriatic and 76 controls) had all data for the hsCRP and atherosclerosis analysis. The control and psoriasis cohorts had similar age, gender, and race distribution, while differences in BMI, smoking status, hypertension, dyslipidemia, LDL, HDL, and hsCRP were present (Table 1). Psoriasis patients had a lengthy history of disease, 19.4 ± 13.3 years, and an average BSA of 14.3 ± 18.8 (Table 1). Psoriatic arthritis affected 24.6 % of the enrolled patients, which is representative of the frequency of psoriatic arthritis in most tertiary care centers [41]. Greater than 90 % of participating patients had plaque type psoriasis with the remainder having either palmo-plantar, inverse or localized pustular psoriasis. There were no patients in this study with generalized pustular psoriasis, erythrodermic psoriasis or with psoriatic arthritis in the absence of any cutaneous disease. Topical treatments, though not necessarily exclusive, were the most frequently utilized therapy (Table 1). The frequency of systemic treatment was 36 % (not shown).

**Table 1:** Cohort demographics and disease characteristics

Demographics	Psoriasis (N = 207)	Controls (N = 76)	p-value
	Mean ± SEM or N (%)	Mean ± SEM or N (%)	
Age (years)	47.8 ± 1.0	48.8 ± 2.0	0.651
Gender (male)	114 (55.1 %)	40 (52.6 %)	0.715
Race (not Caucasian)	24 (11.6 %)	9 (11.8 %)	0.954
*Characteristics of interest*			
BMI (kg/m^2^)	30.9 ± 0.5	28.9 ± 0.1	0.025
Current smoker	78 (37.7 %)	14 (18.4 %)	0.002
Dyslipidemia	110 (53.1 %)	28 (36.8 %)	0.022
Systolic blood pressure	131 ± 1.3	123 ± 2.2	0.002
Fasting blood glucose (mg/dL)	94.7 ± 1.7	91.6 ± 1.4	0.184
HDL (mg/dL)	51.1 ± 1.0	55.9 ± 1.5	0.009
Hypertension	95 (45.9 %)	19 (25.0 %)	0.001
hsCRP	5.6 ± 0.7	3.3 ± 1.0	0.003
LDL (mg/dL)	121.2 ± 2.5	110.4 ± 3.9	0.027
Statin therapy	44 (21.3 %)	16 (21.1 %)	0.97
Total cholesterol (mg/dL)	197.4 ± 3.0	188.9 ± 4.7	0.128
Triglyceride (mg/dL)	125.9 ± 5.4	115.2 ± 11.3	0.392
Waist circumference (in)	40.2 ± 0.5	38.4 ± 0.6	0.028
Coronary artery calcium (CAC)	159 ± 32	130 ± 52	0.649
10 year Framingham risk score	5.8 ± 0.4	4.8 ± 0.8	0.235
*Psoriasis specific*			
BSA	14.3 ± 1.3	N/A	ND
PASI	10 ± 0.7	N/A	ND
PGA	2.2 ± 0.1	N/A	ND
Psoriasis arthritis	51 (24.6 %)	N/A	ND
Psoriasis duration (years)	19.4 ± 1.0	N/A	ND
*Psoriasis treatment(s)*			
Biologics	49 (23.7 %)	N/A	ND
Cyclosporine	5 (2.4 %)	N/A	ND
Methotrexate	13 (6.3 %)	N/A	ND
Narrow band UVB	13 (6.3 %)	N/A	ND
Oral retinoids	7 (3.4 %)	N/A	ND
Topicals	96 (46.4 %)	N/A	ND

Both the psoriasis and control cohorts with a history of hypertension had a similar frequency of non-treatment (Additional file 1: Table S1), defined as not taking any hypertensive medications. Similar findings were present with regard to patients with dyslipidemia (Additional file 1: Table S1), defined as not receiving lipid altering medication (statins, bile acid sequestrants, nicotinic acid, or fibric acid). To ascertain whether our results could be driven by the presence of psoriatic arthritis, analyses were performed with all patients as well as after removing the psoriatic arthritis patients. We found that in subjects with psoriasis only, both untreated hypertension and untreated dyslipidemia were significantly different between psoriasis patients and controls (Additional file 1: Table S2). We categorized subjects with dyslipidemia into high total cholesterol, high LDL or low HDL sub-cohorts using threshold defined by the National Cholesterol Education Program Adult Treatment Panel III (NCEPATP3) (Additional file 1: Table S3). There was no evidence of a difference in untreated dyslipidemia between each of the psoriasis and control sub-cohorts but when we performed this analysis after removing the patients with psoriatic arthritis, patients with an untreated HDL <40 differed from the control patients (Additional file 1: Table S4).

### Psoriasis patients showed higher prevalence of atherosclerosis

**Fig. 1:**
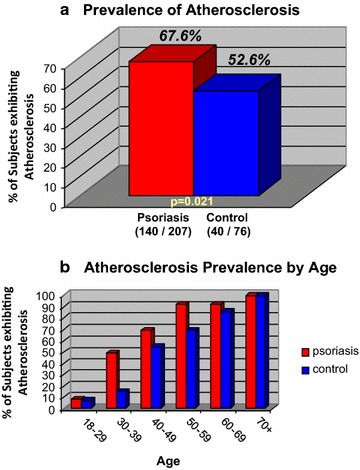
Atherosclerosis prevalence. The prevalence of atherosclerosis in the psoriasis cohort relative to controls was assessed using one or more correlated evidence of atherosclerotic disease, therefore the total number of psoriasis and control patients with atherosclerosis was 140/207 (67.6 %) and 40/76 (52.6 %), respectively (**a**). A breakdown of atherosclerosis by age revealed an excess of atherosclerotic disease in younger psoriasis age groups (of 30–39 year-olds, 49 % of psoriasis patients had atherosclerotic disease versus 15 % of controls in the same age group (**b**)

Ninety-two psoriasis patients had positive CAC scores, 83 had carotid plaque, 87 had a CIMT > 75 percentile and 5 had coronary stents, while 26 controls had positive CAC scores, 23 had carotid plaque, 25 had a CIMT > 75 percentile, and 1 had a coronary stent. One or more of these findings were considered evidence for atherosclerosis, thus the number of psoriasis and controls with atherosclerosis was 140/207 (67.6 %) and 40/76 (52.6 %), respectively (Fig. 1a). The unadjusted odds of atherosclerosis was 88 % higher in psoriasis patients than controls [95 % CI (1.10, 3.21); p = 0.0210; Table 2]. This association was substantially more pronounced after adjusting for age [adjusted OR 3.23; 95 % CI (1.53, 6.80); p = 0.002; Table 2], likely due to an excess of atherosclerosis in younger psoriasis age groups (of 30–39 year-olds, 49 % of psoriasis patients had atherosclerosis versus 15 % of controls in the same age group; Fig. 1b). The association between psoriasis and prevalence of atherosclerosis remained significant after adjustment for gender, race, BMI, smoking status, hypertension, HDL, and hsCRP [adjusted OR 2.67; 95 % CI (1.2, 5.92); p = 0.016; Table 2]. Models that further controlled for additional variables maintained the association: OR 2.76, p = 0.0174 when controlling for fasting blood glucose level s(FBG), and OR 2.60, p = 0.0332 when controlling for LDL and current statin therapy. An analysis of psoriasis patients only using psoriasis severity measured by PASI, PGA, or BSA, failed to establish an association with atherosclerosis (Additional file 1: Table S5) and when this analysis was performed after removing all patients with psoriatic arthritis, the results were unchanged (not shown).

**Table 2:** Association between psoriasis and presence of atherosclerotic disease

Model	Adjusted odds ratio of atherosclerotic disease comparing psoriasis to control patients (95 % CI)	p-value
Presence of psoriasis	1.88 (1.10, 3.21)	0.0210
Presence of psoriasis + age	3.23 (1.53, 6.80)	0.0020
Presence of psoriasis + age + gender	3.18 (1.51, 6.69)	0.0023
Presence of psoriasis + age + gender + race	3.18 (1.51, 6.70)	0.0023
Presence of psoriasis + age + gender + race + BMI	2.93 (1.38, 6.23)	0.0051
Presence of psoriasis + age + gender + race + BMI + current smoker	2.66 (1.23, 5.77)	0.0128
Presence of psoriasis + age + gender + race + BMI + current smoker + hypertension	2.54 (1.16, 5.53)	0.0193
Presence of psoriasis + age + gender + race + BMI + current smoker + hypertension + HDL	2.72 (1.23, 6.03)	0.0138
Presence of psoriasis + age + gender + race + BMI + current smoker +hypertension + HDL + hsCRP	2.67 (1.20, 5.92)	0.0161

Atherosclerosis defined as CAC score ≥1 (or coronary stent), or right or left CIMT >75th percentile, or the presence of carotid plaque

### Association of hsCRP with psoriasis severity

hsCRP was 71 % higher in psoriasis patients compared to controls [unadjusted geometric mean ratio 1.71; 95 % CI (1.20, 2.44); p = 0.0030; Table 3]. This association was not as strong, but still significant after adjusting for age, gender, race, BMI, and smoking status [adjusted geometric mean ratio 1.35; 95 % CI (1.00, 1.82); p = 0.0478; Table 3]. However, after adjustment for hypertension, HDL, and presence of atherosclerosis, a non-significant trend between psoriasis and hsCRP was evident [adjusted geometric mean ratio 1.28; 95 % CI (0.94, 1.74); p = 0.12; Table 3]. Adjustment for additional variables failed to show a significant association [adjusted geometric mean ratios 1.22 (p = 0.2112)] when controlling for FBG, and 1.16 (p = 0.3474) when controlling for LDL and current statin therapy].

**Table 3:** Association between psoriasis and hsCRP

Model	Adjusted geometric mean ratio of hsCRP comparing psoriasis to control patients (95 % CI)	p-value
Presence of psoriasis	1.71 (1.20, 2.44)	0.0030
Presence of psoriasis + age	1.74 (1.22, 2.47)	0.0020
Presence of psoriasis + age + gender	1.75 (1.24, 2.48)	0.0016
Presence of psoriasis + age + gender + race	1.75 (1.24, 2.48)	0.0016
Presence of psoriasis + age + gender + race + BMI	1.43 (1.06, 1.91)	0.0180
Presence of psoriasis + age + gender + race + BMI + current smoker	1.35 (1.00, 1.82)	0.0478
Presence of psoriasis + age + gender + race + BMI + current smoker + hypertension	1.32 (0.97, 1.78)	0.0769
Presence of psoriasis + age + gender + race + BMI + current smoker + hypertension + HDL	1.31 (0.97, 1.78)	0.0830
Presence of psoriasis + age + gender + race + BMI + current smoker + hypertension + HDL + atherosclerosis	1.28 (0.94, 1.74)	0.1180

Through analysis of psoriasis patients only, we found a statistically significant association between BSA and hsCRP [unadjusted geometric mean ratio per 15 unit increase in BSA 1.29; 95 % CI (1.11, 1.49); p = 0.0008; Table 4]. This association was not as pronounced after adjusting for age, gender, race, BMI, current smoking status, history of hypertension, HDL, and presence of atherosclerosis [adjusted geometric mean ratio 1.21; 95 % CI (1.07, 1.37); p = 0.0022; Table 4]. Although adjustment for additional variables maintained the association: adjusted geometric mean ratio = 1.013 (p = 0.0030) when controlling for FBG, and 1.013 (p = 0.0035) when controlling for LDL and current statin therapy. We also found an association between PASI and hsCRP [adjusted geometric mean ratio per 10 unit increase in PASI: 1.28; 95 % CI (1.11, 1.48); p = 0.0006] using the same modeling as for BSA. Models that further controlled for additional variables maintained the association: adjusted geometric mean ratio 1.026 (p = 0.0012) when controlling for FBG, and 1.025 (p = 0013) when controlling for LDL and current statin therapy. An association between PGA and hsCRP was also noted in the same modeling test (adjusted geometric mean ratio per 1 unit increase in PGA 1.33; 95 % CI (1.15, 1.55); p = 0.0002; Table 4). Models that further controlled for additional variables within the PGA:hsCRP relationship maintained the association: adjusted geometric mean ratio 1.28 (p = 0.0052) when controlling for FBG, and 1.27 (p = 0054) when controlling for LDL and current statin therapy.

**Table 4:** Association between psoriasis severity and hsCRP

Model	Adjusted geometric mean ratio of hsCRP for every 15 % increase in BSA (95 % CI)	p-value	Adjusted geometric mean ratio of hsCRP for every 10 unit increase in PASI (95 % CI)	p-value	Adjusted geometric mean ratio of hsCRP for every 1 unit increase in PGA (95 % CI)	p-value
Psoriasis severity	1.29 (1.11, 1.49)	0.0008	1.36 (1.15, 1.62)	0.0001	1.42 (1.18, 1.71)	0.0002
Psoriasis severity + age	1.31 (1.13, 1.51)	0.0003	1.39 (1.17, 1.65)	0.0001	1.44 (1.21, 1.73)	0.0001
Psoriasis severity +age + gender	1.31 (1.14, 1.52)	0.0002	1.40 (1.18, 1.66)	0.0001	1.45 (1.21, 1.74)	<0.0001
Psoriasis severity +age + gender + race	1.32 (1.14, 1.52)	0.0002	1.41 (1.18, 1.67)	0.0001	1.45 (1.21, 1.74)	0.0001
Psoriasis severity +age + gender + race + BMI	1.23 (1.09, 1.39)	0.0010	1.31 (1.13, 1.51)	0.0002	1.35 (1.16, 1.57)	0.0001
Psoriasis severity + age + gender + race +BMI + current smoker	1.22 (1.08, 1.38)	0.0013	1.30 (1.13, 1.50)	0.0003	1.33 (1.14, 1.55)	0.0002
Psoriasis severity + age + gender + race + BMI + current smoker + hypertension	1.21 (1.07, 1.37)	0.0018	1.29 (1.12, 1.49)	0.0005	1.33 (1.15, 1.55)	0.0002
Psoriasis severity + age + gender + race + BMI + current smoker + hypertension + HDL	1.21 (1.08, 1.37)	0.0018	1.29 (1.12, 1.49)	0.0005	1.33 (1.15, 1.55)	0.0002
Psoriasis severity + age + gender + race + BMI + current smoker + hypertension + HDL + atherosclerosis	1.21 (1.07, 1.37)	0.0022	1.28 (1.11, 1.48)	0.0006	1.33 (1.15, 1.55)	0.0002

The Hosmer and Lemeshow goodness-of-fit tests for the logistic models yielded values ranging between 0.10 and 0.99, thus providing no indication of lack of fit of any of the models. Residuals did not suggest important departures from linearity. A sensitivity analysis that excluded the patients with a medical history of cardiovascular disease (n = 15) yielded results consistent with those presented here (data not shown).

## Discussion

We have shown that even after adjusting for multiple confounding factors, a tertiary care cohort of psoriasis patients have a 2.67-fold higher odds of having atherosclerosis compared to controls. The most striking finding was that after age stratification, almost half (49 %) of patients with psoriasis aged 30–39 years had evidence of subclinical atherosclerosis as compared to 15 % of controls. These findings are suggestive that psoriasis may contribute to the inflammatory cascade of atherosclerosis and that the younger a patient develops psoriasis, the higher the CVD risk. These findings are consistent with recent observations from preclinical models systems, wherein chronic skin-contained inflammation, but not acute, promoted the acceleration of arterial thrombosis [19] and [20]. In addition, other investigators have also reported observational studies demonstrating relatively higher measures of association with CV events in younger individuals [25, 42–44]. Psoriasis sufferers with long-standing and more severe disease, as well as those with joint involvement likely have a greater systemic inflammatory burden that may increase their likelihood of distant effect on the vascular system. Inflammation plays a crucial role in the initiation and promotion of atherosclerosis [18, 45, 46]. The immunologic commonalities of inflammation, linking psoriasis and atherosclerosis include infiltrating T-cells, macrophages, monocytes, dendritic cells, and mast cells in psoriatic plaques, and a similar composition of cells in atherosclerotic plaques [47–49]. A similar pattern of CD4^+^ T-cell activation through antigen presenting dendritic cells stimulate the proliferation of CD8^+^ T-cells, with activity of the T-helper 1 phenotype inflammation that prevails in both psoriasis and atherosclerosis [2, 4, 47, 48, 50, 51]. This may lead to cyclic inflammation through continuous activation and re-activation of T-cells and macrophages and their ensuing cytokines that result in systemic inflammation mechanisms common to psoriasis and atherosclerosis [2, 4, 48]. Further support of this idea comes from results demonstrating that effective treatment of psoriasis may improve endothelial cell function [52].

We did not find evidence of an association between severity of psoriasis, as measured by PASI, PGA, or BSA, and the presence of atherosclerosis in this study where psoriasis patients were included without regard to their current treatment type, dosage, or duration. We did not observe a causal role for psoriatic arthritis in our findings; when patients with psoriatic arthritis were removed from our analyses there were more patients with psoriasis only with untreated hypertension and untreated HDL <40 than observed in the controls cohort.

Although other studies have examined vascular imaging techniques such as CIMT, FMD, or CAC scores to evaluate CVD and reported associations with psoriasis [53–55], our study utilized a more comprehensive technique for assessing atherosclerosis, with a more analogous control cohort, and adjusted for more confounding factors than previous studies. We used a multi-vessel, multi-site, cumulative approach to detect the spectrum of atherosclerosis from the sub-clinical origins through clinical interventions in all subjects, while utilizing a control cohort recruited from the same clinic. Two other studies have examined CAC scores in psoriasis patients. Both of these studies, like ours, demonstrated increased odds of CAC in psoriasis patients versus controls (after CVD risk factor adjustment), although our study had an even higher percentage of controls with CAD (34 versus 28 versus 4 %) [54, 56]. However, these studies examined smaller numbers of patients than our current study. In addition, only one other study has used multiple imaging modalities in the same patient population [56].

Several other studies have evaluated CIMT in psoriasis patients. All of these studies demonstrated increased CIMT in psoriasis versus controls, which is consistent with our results. In several of these studies, IMT correlated with psoriasis severity [53, 57]. Studies involving only patients with psoriatic arthritis, which has a greater burden of systemic inflammation than psoriasis, have established an association with atherosclerosis through evaluation of CIMT and carotid plaque [58–60]. These surrogate markers have been validated as useful, non-invasive techniques for evaluating atherosclerosis and stratifying risk of future CVD events [38–40, 61, 62].

While our data demonstrated a trend toward elevated hs-CRP in psoriasis patients compared to controls, several other studies have demonstrated statistically significant elevations of hs-CRP levels in psoriasis patients [63, 64]. The findings of Troitzsch et al. [63], may be limited due to their study relying upon a population-based approach that lacks details regarding psoriasis severity or duration, and the Usta et al. [64] report reflects a small study that included patients with very mild disease. This suggests that systemic inflammation, especially in psoriasis patients, is multi-factorial and the driving influence as determined by hsCRP, is shared. Inflammation plays a central role in atherosclerosis, and CRP is a critical factor in inflammation [18, 28]. We show that serum CRP, detected by the high sensitivity test (hs-CRP, one marker of systemic inflammation) is elevated in psoriasis patients compared to controls even after adjusting for several variables including age, gender, race, BMI, and current smoking status. However, the association was lost after adjusting for hypertension, HDL, and presence of atherosclerosis. These factors individually influence inflammation and hsCRP levels [65–69]. Furthermore, the cross-sectional design of this study which included stable, well-controlled psoriasis patients receiving potent treatments may have decreased levels of systemic inflammation and potentially underestimated the significance of this relationship. Additionally, the study may not have been sufficiently powered to detect significant differences between psoriasis severity and atherosclerosis.

Finally, we have recently demonstrated elevated circulating intermediate monocytes (CD14^++^, CD16^+^; Mon2) in psoriasis patients; along with increases in the formation of monocyte-aggregates; thus this cell population may provide an additional surrogate outcome measure for predicting CVD in a psoriasis patient cohort that is more refined [70]. These cells correlated to PASI and have previously been demonstrated to be elevated in high risk non-psoriasis CVD patients [71–74]. Whether this population of cells may be capable of serving as a surrogate marker of skin-mediated promotion of adverse cardiac events in psoriasis patients, and how it changes with successful resolution of psoriasis following systemic treatment, remains to be determined. However, it is clear that current measures of psoriasis severity may lack the precision to address the severity of psoriasis and prevalence of atherosclerosis. Thus developing alternative measures of monitoring patient response to therapy is worthwhile.

## Limitations

This is a cross-sectional cohort study of a tertiary care group of psoriasis patients and is subject to the limitations of this design. Since these patients are recruited from a tertiary care center, they may represent a more severe psoriasis cohort than average. In particular, patients were eligible for enrollment regardless of disease severity, duration, treatment type or duration. Compared to controls, our psoriasis patients had higher BMI, LDL, triglycerides, prevalence of smoking, prevalence of hypertension, and lower HDL. These variables could be confounders of the association between psoriasis (or psoriasis severity) and hsCRP as well as psoriasis (or psoriasis severity) and atherosclerosis. The methods of assessing psoriasis severity may be problematic because of floor and ceiling effects. Although multivariable analyses attempted to adjust for these confounders, our results may be biased due to residual confounding. In addition, some data elements were missing and these subjects were excluded. Consequently, any inference could be biased due to potential selection bias stemming from missing data. However, because missing data occurred in less than 5 % of our patients, this is less likely to be cause for concern. Additionally, our psoriasis patients tended to have more severe disease and therefore our results may be more generalizable to patients with more severe disease. Finally, it should be noted that a cross sectional design cannot establish direction of an association.

## Conclusions

A tertiary care cohort of psoriasis patients have a high prevalence of early atherosclerosis and elevated levels of serum CRP, detected by the high sensitivity test (hs-CRP, one marker of systemic inflammation), and psoriasis was a risk factor for the presence of atherosclerosis even after adjustment of key confounding clinical factors. Psoriasis may contribute to development of atherosclerosis due to an accelerated systemic inflammatory cascade resulting in increased risk of CVD and cardiovascular events. Future research should focus on whether effective treatment of psoriasis reduces the risk of atherosclerosis, CVD, morbidity, and mortality.

## Additional file

10.1186/s12967-016-0947-0 Additional supplementary tables.

## Abbreviations

CVD: cardiovascular disease
CAC: coronary artery calcium
CIMT: carotid intima-media thickness
hsCRP: high sensitivity C-reactive protein
PUVA: psoralen plus ultraviolet A light
PASI: psoriasis area severity index
PGA: Physician’s Global Assessment
BSA: body surface area
