# Supplementary material for: Visualization of atherosclerosis as detected by coronary artery calcium and carotid intima-media thickness reveals significant atherosclerosis in a cross-sectional study of psoriasis patients in a tertiary care center
Source: J Transl Med. 2016 Jul 22;14:217. doi: 10.1186/s12967-016-0947-0 (PMC4957305; doi:10.1186/s12967-016-0947-0)
Supplement: Supplementary file 1 — 10.1186/s12967-016-0947-0 Additional supplementary tables. [file 12967_2016_947_MOESM1_ESM.docx]

| **Table S1. Untreated co-morbidities in all patients** | | | |
| --- | --- | --- | --- |
| **Co-morbidity** | **Psoriasis patients with co-morbidity** | **Control patients with co-morbidity** | **p-value** |
| Untreated hypertension^1^ | 35/95 = 36.8% | 10/19 = 52.6% | 0.30 |
| Untreated dyslipidemia^2^ | 60/110 = 54.5% | 12/28 = 43.0% | 0.37 |
| ^1^Patients with blood pressure ≥140 or ≥90 at exam and not taking anti-hypertensive treatment.  ^2^Patients with LDL ≥ 160, or total cholesterol ≥ 240, or HDL < 40 from fasting serum sample (lab calculated via Friedewald) and not taking lipid altering medication including statins, bile acid sequestrants, nicotinic acid, and fibric acid. Note that these thresholds were determined by ATPIII. | | | |

| **Table S2. Untreated co-morbidities after removing psoriatic arthritis patients** | | | | | | | | | | | |
| --- | --- | --- | --- | --- | --- | --- | --- | --- | --- | --- | --- |
| **Co-morbidity** | **Psoriasis patients with co-morbidity** | | | | **Control patients with co-morbidity** | | | | **p-value** | | |
| Untreated hypertension^1^ | 68/156 = 43.6% | | | | 19/76 = 25.0% | | | | 0.0061 | | |
| Untreated dyslipidemia^2^ | 54/156 = 34.6% | | | | 15/76 = 19.7% | | | | 0.0200 | | |
| ^1^Patients with blood pressure ≥140 or ≥90 at exam and not taking anti-hypertensive treatment.  ^2^Patients with LDL ≥ 160, or total cholesterol ≥ 240, or HDL < 40 from fasting serum sample (lab calculated via Friedewald) and not taking lipid altering medication including statins, bile acid sequestrants, nicotinic acid, and fibric acid. Note that these thresholds were determined by ATPIII. | | | | | | | | | | | |
| **Table S3. Untreated abnormal values** | | | | | | | | | | | |
| **Abnormal value** | | **Psoriasis patients with abnormal value** | | | | **Control patients with abnormal value** | | | | **p-value** | |
| Untreated LDL ≥ 160^1^ | | 11/13 = 85% | | | | 5/6 = 83% | | | | 0.54 | |
| Untreated total cholesterol ≥ 240^2^ | | 29/32 = 91% | | | | 8/9 = 89% | | | | 0.63 | |
| Untreated HDL < 40^3^ | | 33/44 = 75% | | | | 5/7 = 71% | | | | 0.79 | |
| ^1^Patients with LDL ≥ 160 and no current use of lipid altering medication including statins, bile acid sequestrants, nicotinic acid, fibric acid. Note that this high threshold was determined by ATPIII.  ^2^Patients with total cholesterol ≥ 240 and no current use of lipid altering medication including statins, bile acid sequestrants, nicotinic acid, fibric acid. Note that this high threshold was determined by ATPIII.  ^3^Patients with HDL < 40 and no current use of lipid altering medication including statins, bile acid sequestrants, nicotinic acid, fibric acid. Note that this low threshold was determined by ATPIII. | | | | | | | | | | | |
| **Table S4. Untreated abnormal values after removing psoriatic arthritis patients** | | | | | | | | | | | |
| **Abnormal value** | | **Psoriasis patients with abnormal value** | | | | **Control patients with abnormal value** | | | | **p-value** | |
| Untreated LDL ≥ 160^1^ | | 19/156 = 12.2% | | | | 6/76 = 7.9% | | | | 0.3232 | |
| Untreated total cholesterol ≥ 240^2^ | | 22/156 = 14.1% | | | | 9/76 = 11.8% | | | | 0.6348 | |
| Untreated HDL < 40^3^ | | 33/156 = 21.2% | | | | 7/76 = 9.2% | | | | 0.0238 | |
| ^1^Patients with LDL ≥ 160 and no current use of lipid altering medication including statins, bile acid sequestrants, nicotinic acid, fibric acid. Note that this high threshold was determined by ATPIII.  ^2^Patients with total cholesterol ≥ 240 and no current use of lipid altering medication including statins, bile acid sequestrants, nicotinic acid, fibric acid. Note that this high threshold was determined by ATPIII.  ^3^Patients with HDL < 40 and no current use of lipid altering medication including statins, bile acid sequestrants, nicotinic acid, fibric acid. Note that this low threshold was determined by ATPIII. | | | | | | | | | | | |
| **Table S5. Association between psoriasis severity and prevalence of atherosclerotic disease** | | | | | | | | | | | |
| Model | Adjusted  odds ratio of atherosclerotic disease  for every 15% increase in BSA  (95% CI) | | p-value | Adjusted  odds ratio of atherosclerotic disease  for every 10 unit increase in PASI  (95% CI) | | | p-value | Adjusted  odds ratio of atherosclerotic disease  for every 1 unit increase in PGA  (95% CI) | | | p-value |
| Psoriasis severity | 1.07 (0.84, 1.36) | | 0.605 | 1.07 (0.80, 1.42) | | | 0.646 | 1.01 (0.76, 1.36) | | | 0.922 |
| Psoriasis severity +age | 1.22 (0.92, 1.63) | | 0.174 | 1.30 (0.91, 1.85) | | | 0.150 | 1.16 (0.81, 1.67) | | | 0.407 |
| Psoriasis severity +age+gender | 1.22 (0.91, 1.63) | | 0.190 | 1.29 (0.90, 1.84) | | | 0.164 | 1.16 (0.81, 1.66) | | | 0.430 |
| Psoriasis severity +age+gender+race | 1.22 (0.91, 1.64) | | 0.191 | 1.30 (0.91, 1.86) | | | 0.164 | 1.15 (0.80, 1.66) | | | 0.437 |
| Psoriasis severity +age+gender+race +BMI | 1.18 (0.87, 1.58) | | 0.284 | 1.24 (0.86, 1.77) | | | 0.245 | 1.10 (0.76, 1.59) | | | 0.604 |
| Psoriasis severity +age+gender+race +BMI+current smoker | 1.15 (0.86, 1.55) | | 0.353 | 1.20 (0.84, 1.72) | | | 0.311 | 1.05 (0.72, 1.54) | | | 0.788 |
| Psoriasis severity +age+gender+race +BMI+current smoker +hypertension | 1.14 (0.85, 1.54) | | 0.385 | 1.19 (0.83, 1.71) | | | 0.345 | 1.05 (0.72, 1.54) | | | 0.782 |
| Psoriasis severity +age+gender+race +BMI+current smoker +hypertension+HDL | 1.15 (0.85, 1.55) | | 0.375 | 1.20 (0.83, 1.73) | | | 0.327 | 1.04 (0.71, 1.53) | | | 0.834 |
| Psoriasis severity +age+gender+race +BMI+current smoker +hypertension+HDL +hsCRP | 1.13 (0.83, 1.53) | | 0.452 | 1.18 (0.82, 1.70) | | | 0.384 | 1.02 (0.70, 1.50) | | | 0.913 |
